# Exploring the reasons for the large density of triplex-forming oligonucleotide target sequences in the human regulatory regions

**DOI:** 10.1186/1471-2164-7-63

**Published:** 2006-03-27

**Authors:** Josep Ramon Goñi, Juan Manuel Vaquerizas, Joaquin Dopazo, Modesto Orozco

**Affiliations:** 1 Molecular Modeling and Bioinformatics Unit. Institut de Recerca Biomèdica. Parc Científic de Barcelona. Josep Samitier 1-5. Barcelona 08028. Spain; 2 Department of Bioinformatics, Centro de Investigación Príncipe Felipe, Avda. Autopista del Saler 16, Valencia, 46013, Spain; 3 Functional Genomics Node, Instituto Nacional de Bioinfomatica, Centro de Investigación Príncipe Felipe, Avda. Autopista del Saler 16, Valencia 46013, Spain; 4 Departament de Bioquímica i Biología Molecular. Facultat de Química. Universitat de Barcelona. Martí i Franquès 1. Barcelona 08028. Spain; 5 Protein Structure and Modeling Node. Instituto Nacional de Bioinfomàtica. Genoma España. Parc Científic de Barcelona. Josep Samitier 1-5. Barcelona 08028. Spain

## Abstract

**Background:**

DNA duplex sequences that can be targets for triplex formation are highly over-represented in the human genome, especially in regulatory regions.

**Results:**

Here we studied using bioinformatics tools several properties of triplex target sequences in an attempt to determine those that make these sequences so special in the genome.

**Conclusion:**

Our results strongly suggest that the unique physical properties of these sequences make them particularly suitable as "separators" between protein-recognition sites in the promoter region.

## Background

**Figure 1:**
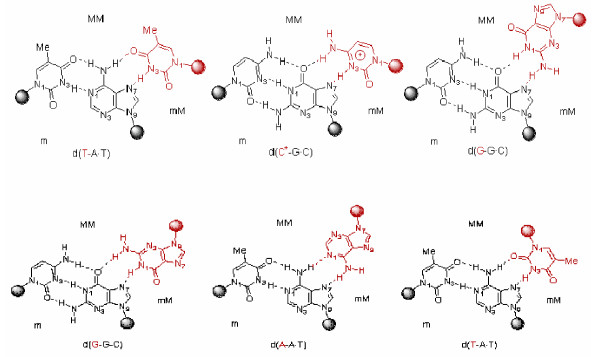
Schematic representation of parallel and anti-parallel triads present in triplexes.

DNA triplexes [1] are formed when a duplex containing a poly-purine track is recognized by single-stranded polynucleotide (noted as the triplex-forming oligonucleotide; TFO). The third strand interacts through the major groove of the duplex, thereby making specific hydrogen bond interactions with the Watson-Crick purines [2,3]. The TFO can be DNA, RNA or different oligonucleotides with modifications in either their nucleobases or phosphoribose backbone [4]. Two types of triplexes have been described on the basis of the orientation of the TFO with respect to the central polypurine track: i) parallel triplexes and ii) anti-parallel triplexes. The parallel triplex is characterized by Hoogsteen hydrogen bonds between the TFO (typically pyrimidine-rich) and the central Watson-Crick purine [see Figure 1], while the anti-parallel triplexes show reverse-Hoogsteen hydrogen bonds and the TFO is purine-rich [see Figure 1]. Parallel triplexes are believed to be more stable than the anti-parallel ones in normal laboratory conditions, but the situation can reverse in physiological environments, especially when the target duplexes contain a poly-G track [2-5].

The presence of a TFO in the major groove of the duplex leads to major distortions in the capacity of the target duplex to be recognized by specific proteins [2,6,7]. This produces major changes in the functionality of the target duplex, which has been used for biotechnological and biomedical purposes [2,3,8-10]. Thus, modified TFOs containing suitable chemical compounds have been used to develop artificial nucleases [11,12], to induce recombination in mammalian cells [13], and to trigger mutations in target DNA [13-16]. In all these cases, the formation of the triplex guides the active chemical compound to the proper position in the target genome. Unmodified TFOs increase the rate of mutations at the triplex target sequence (TTS), which opens the possibility for knocking down genes [9,16,17]. Triplex formation inhibits mRNA synthesis [2,8,9,18-23] when the TTS is located at a regulatory region. Furthermore, when the triplex is formed in the middle of a gene, mRNA elongation is stopped just before the TTS, which indicates that triplex binding is strong enough to displace complex transcriptional machinery [24,25]. These two findings open up the possibility to use TFOs as "anti-gene" drugs. These pharmacological agents would have the capacity to specifically arrest the transcription of pathological genes, thus leading to an intense and targeted therapeutic action [3,8-10]. However, despite their promise, anti-gene therapies still face many technical problems [2,3] and the density and location of TTSs in human genes is unclear.

In a recent paper, we explored the presence of TTSs (polypurine tracts which are expected to lead to stable triplexes in physiological conditions) in the human genome [26]. Our analysis showed that these sequences are vastly over-represented when compared to what randomness predicts. Interestingly, the largest relative concentration of TTSs occurs in the upstream regulatory region (especially at the proximal promoter region: 100 nts upstream) [26]. Recent studies by our group (Goñi *et al*. Unpublished results) show that these trends are common to many other organisms, from mammals to procaryotes, indicating that many genes may be targets for triplex formation (Goñi *et al*. Unpublished results). However, this interesting finding raises an intriguing question: why are TTSs so abundant in crucial regions for the control of genome function?

Here we present an extensive descriptive analysis of TTSs in the human genome in an attempt to elucidate why these sequences are so abundant in regulatory regions. Our results indicate that the unique physical properties of TTSs may explain this overpopulation.

## Results and discussion

**Figure 2:**
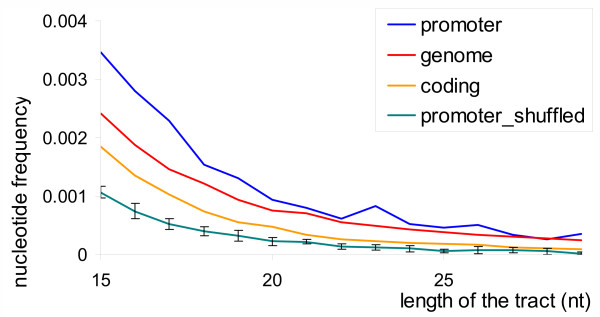
Frequencies of nucleotides forming part of TTSs for different lengths in the human genome, coding and promoter regions (100 nucleotides upstream). Values represented here correspond to total population from the human genome and accordingly do not have error bars associated. Random promoter expected values and its deviation is computed generating 10 sets of sequences shuffling our promoter collection.

As proposed in previous studies using an older genomic data base [26], TTSs are largely over-represented in human genome with respect to a background model such as that described in reference [[26]; see Figure 2]. This over-representation is particularly noticeable when considering the proximal promoter region (100 nts upstream), where a considerable density of large TTSs are found. Note that the over-representation is clear irrespective of the random model used (for the shake of simplicity only few random models are shown in Figure 2; the rest are displayed in supplementary material [see Additional file 1]) and that the statistical significance of the difference is demonstrated by Clover calculations (p < 10^-20 ^; [see *Methods*]).

**Figure 3:**
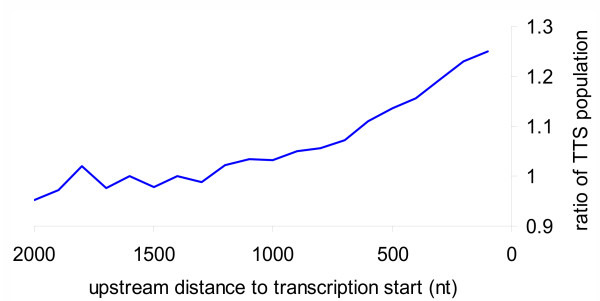
Ratio of TTSs (lengths from 10 to 25 nts) in promoter regions (from 2000 to 100 nts upstream) with respect to average TTS population in the human genome. Once again data is taken for the entire genome and correspond to absolute values, without associated errors.

**Figure 4:**
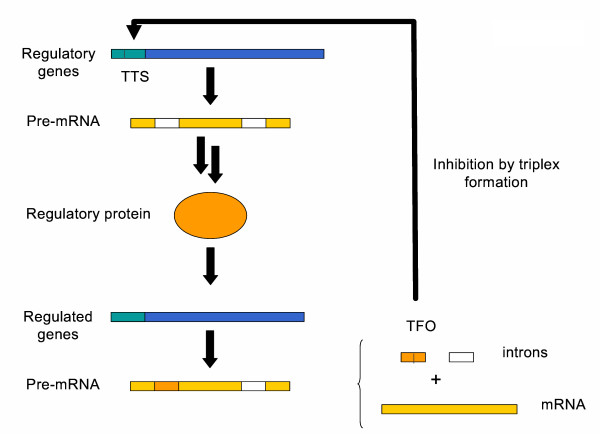
Putative feed-back regulatory mechanism for the control of gene function on the basis of the inhibition of regulatory genes by triplex formation between TTSs in the promoter of regulatory genes and the TFOs in the introns of regulated genes.

Very interestingly, the over-representation of TTSs in the promoter region with respect to the general human genome decreases as more distant promoter regions are considered [see Figure 3]. Clearly, there is an unusually large region with potential to form triplexes in region proximal to transcription origin, which is rich in promoter regions. At first glance, several reasons for this behaviour can be offered: i) triplex formation may be an ancient regulatory mechanism [see Figure 4] for RNA-mediated control of gene expression, ii) target sequences for transcription factors have an overpopulation of TTSs, iii) TTSs have several intrinsic physical properties that are useful for protein binding to DNA.

### Are TTSs part of an ancient DNA auto-regulatory mechanism?

**Figure 5:**
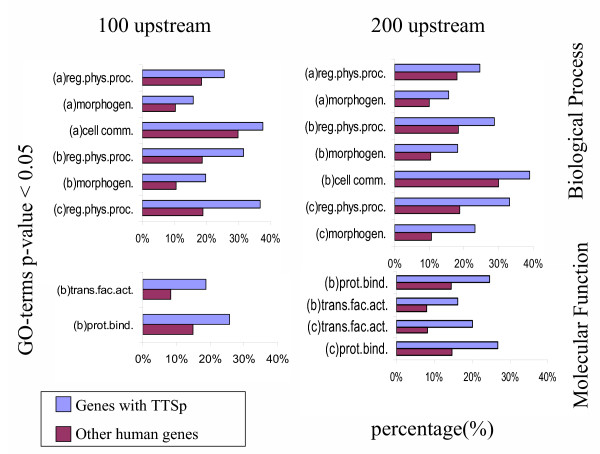
Results of GO analysis of biological processes (upper panels) and molecular functions (bottom panels) of genes with TTS at promoter regions. Analyses were repeated for promoter regions defined by 100 or 200 nts upstream, and considering several TTS lengths (a)15, b)20 and c)25 nts). We show only cases where i) the population is greater than 10% in the set of genes with TTS_p _and ii) the subset of genes with TTS_p _is significantly different p < 0.05 to the background genes using a very strict FDR-adjusted p-test [see *Methods*]. For all the cases shown, the normal p-test is 10^-4 ^to 10^-5^.

Triplex formation is a powerful mechanism by which to modulate gene function, and, when formed in the promoter region, triplexes can knock-out or knock-down a gene. Accordingly, it is feasible that the large number of TTSs at a promoter region is related to regulatory processes. Interestingly, analysis of GO terms using the FATIGO [see *Methods*] program shows that the set of genes with large TTSs (15–20 nts) at promoter regions correspond to a subset of genes which differs significantly (even for the very strict Benjamin-Yekutieli adjusted p-value; [see *Methods*]) from the background. Irrespectively of the length of the TTS (from 15 to 25 nts) and the section of the early promoter region 0–100 or 0–200 nts upstream, genes with TTS_p _are over-enriched with functions in the regulation of physiological processes, and very often are characterized as transcription factors or related protein [see Figure 5]. In fact, TTS in promoters seems to be as determinant of the functionality of genes as the CpG islands [see Additional file 2]. On the basis of this observation, we therefore propose that the presence of large TTS in the promoter region of these genes might provide advantage for the control of the expression of these genes. Furthermore, it is tempting to consider the existence of an RNA-mediated feed-back mechanism [see Figure 4] which controls the expression of the regulatory genes by triplex formation between the TTS at the promoter region of these genes and the TFO present in the intron of the regulated genes. Unfortunately, GO analysis and inspection of *TRANSFAC 8.3 *[see *Methods*] failed to provide evidence connecting transcription factors with TTS at the promoter region with those with the corresponding TFO in introns. In addition, we analysed the cases in which a transcription factor with a TTS in its promoter interacted with a second transcription factor, in order to examine whether the introns of genes regulated by this second factor contained TFOs complementary to the TTSs in the first transcription factor. However, again we did not find any relationship between genes with TTS in the promoter and those with intronic TFOs. Given the low number of cases for which experimental evidence of regulation by transcription factors is available, this negative result cannot be taken as evidence against the RNA-mediated feed-back mechanism proposed.

### Are TTSs rich in transcription factor recognition sites?

**Figure 6:**
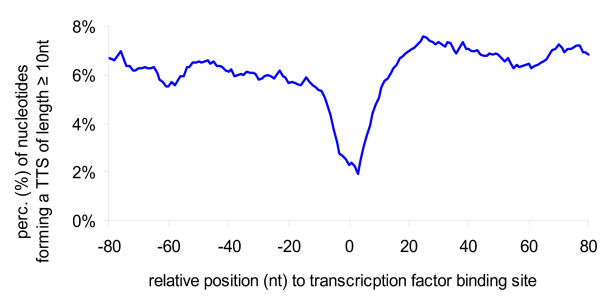
Percentage of nucleotides forming TTS (length equal or greater than 10 nts) at a range of distances from the centre of a transcription factor recognition site. Negative values imply upstream direction and positive values approach the transcription starting point. Calculations were performed considering only the 200 nt early promoter region.

As described in *Methods*, we mapped the TRANSFAC database into the human promoter region (up to 200 nts upstream of transcription origin) and computed the occurrences of nucleotides in long TTSs (length equal or greater than 10) in the promoter region around the transcription factor binding site (TFBS). Sequences which were recognized as targets of transcription factors showed much less probability to be in TTS than neighbouring promoter sequences [see Figure 6]. Furthermore, generation of random sequences (TTS and no-TTS) showed that no-TTS random sequences have a much larger probability to be transcription factor binding sites than TTS random sequences. Overall, we must conclude that even in some cases small (4–6) poly-purine segments might be found in TFBS, TTS as defined here (length equal or greater than 10) are very rarely present in TFBS. That means that the hypothesis that TTSs are over-represented in promoter regions because they contain TFBS can be ruled out.

**Figure 7:**
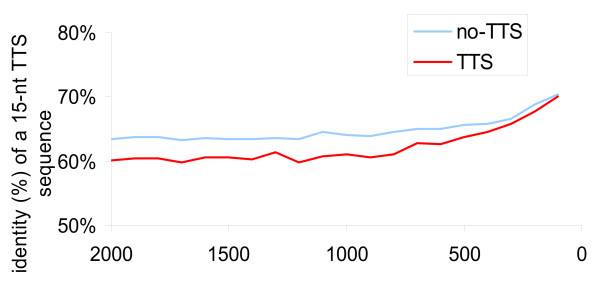
Percentage of human-mouse identity of 15-nt fragments in several promoter regions for TTS and non-TTS segments of the same size in equivalent regions of regulatory region.

Although TTSs do not interact directly with transcription factors, they show a profile of conservation when approaching to the start of transcription similar to that of the whole of non-TTS and of sequences that have been annotated as TFBS [see Figure 7 and Additional file 3]. TTS_p _in the near promoter regions are quite well conserved (even not as conserved as TFBS; [see Additional file 3]) suggesting that they may have an important physiological role that is not related to direct DNA-protein interactions.

### Do TTSs have distinct intrinsic physical properties?

Previous results seem contradictory and somehow difficult to rationalize. Thus, although TTS in promoters (TTS_p_) are over-represented, appear in key genes for the control of physiological processes and are very well conserved, they are not targets for transcription factors. No evidence is found regarding the possibility that TTS acted as an ancient regulatory mechanism, mimicking the functionality of interference RNAs. How can we reconcile all this findings? In our view, the only possibility will be if TTS have some intrinsic physicochemical properties that are useful when present in the promoter region of certain genes.

**Figure 8:**
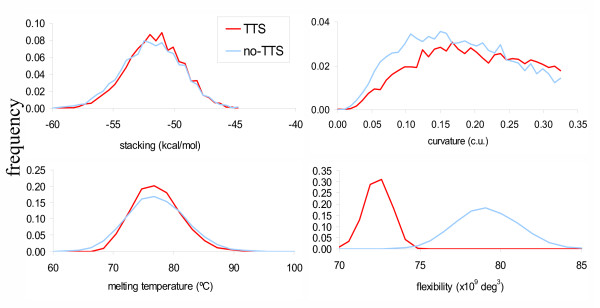
Distribution of selected physical properties of TTSs and random DNA sequences [see *Methods*] taken from human DNA. Analyses were performed on 25-nt fragments; similar results were obtained with shorter segments.

As described in *Methods*, we analyzed several physical-chemical descriptors of DNA in two sets: i) randomly generated TTSs and ii) randomly generated human-like DNA. In general, TTSs displayed average melting temperatures similar to those of normal DNA sequences [see Figure 8], which agrees with the observation that the average stacking energy of TTSs and normal DNA sequences are the same. Thus, TTSs do not introduce bias in the stability of the DNA duplex, which could provide an advantage for the functionality of promoter regions. Curvature analysis using Bolshoy's algorithm indicates that TTSs are significantly more curved than random DNA sequences. Furthermore, analysis of configurational volume [see *Methods*] strongly suggests that TTSs are on average more rigid than normal DNA. These findings strongly support the hypothesis that TTSs at promoter regions can be used as rigid and curved separation signals for transcription factors. It is clear that these physical properties modulate nucleosome positioning and rotational phasing, and several authors have pointed out that polypurine tracks are not well incorporated into the nucleosome [27]. Unfortunately, sequence rules for nucleosome positioning and phasing are, in our hands, not accurate enough to test this hypothesis.

In addition to the possible role of TTSs in the organization of DNA in nucleosomes, when these sequences are present, their unique physicochemical properties have a large impact on the promoter region. Thus, large flexibility is desirable for DNAs that need to bind to proteins, and accordingly deform its structure, but rigidity is useful for the definition of spacing elements that should isolate protein-induced DNA deformability in specific regions of the duplex. The larger curvature is also a desired element, since it can help in the relative positioning of transcription factors in 3-D space, helping then to establish physiologically critical protein-protein interactions. Thus, the presence of TTSs at promoter regions can provide the cell with specific mechanisms, probably in most cases not related to triplex formation, by which to enhance activation/repression of genes that are crucial for the regulation of cellular processes mechanisms.

The results presented in this paper show that, irrespectively of whether the cell now uses or once used a triplex-mediated control mechanism, TTSs in the promoter region are very abundant and that genes with TTS_p _are crucial for the control of cell life. TTSp do not bind to transcription factors, but besides this, they have a conservation profile similar to that of non-TTS segments in promoter regions, including that of sequences recognizing transcription factors. Analysis reported here suggests that the TTS_p _provide the promoter region unique physical properties that can contribute to a better functioning of regulatory proteins. All these results strongly support the notion that triplex-based anti-gene technology is widely applicable in the control of pathologies related to malfunctioning of the regulatory mechanisms of physiological processes.

## Conclusion

Triplex-target sequences (TTS) are over-represented in the human genome. Such an over-representation is especially large when promoter regions 100 to 200 upstream are considered.

Genes with TTS in promoters are over-enriched with functions in the regulation of physiological processes, and very often are characterized as transcription factors or related protein.

TTS are not part of sequences which are directly targeted by transcription factors, but their (human-mouse) conservation profiles suggest that they are important for gene functionality.

TTS are significantly more curved and rigid than normal DNA, which suggests that (in addition to other possible functions) TTS act as spacing fragments which help in the correct positioning of transcription factors.

## Methods

### Genome information

Sequence information of the human genome was taken from the UCSC database [28] version hg17; May 2004; developed by the International Human Genome Mapping Consortium [29]. Annotation of the genes, introns, exons and coding regions considered in this study was obtained from the *refGene *(*refSeq*) collection. To avoid compute multiple times the same locus of the genome, overlapping entries of *refSeq *have been ignored. The set of upstream regions at starting positions 5000, 2000, 1000, 500, 200 and 100 nts upstream of the transcription start of *refSeq *genes were extracted from the *upstream5000 *file of the same data base. The 0–5000 and specially the 0–100 regions are expected to be largely enriched in promoter sequences. USCS also provide the annotation of CpG islands (*CpGisland *file). CpG promoters are those with an overlapping feature on the 200-nt upstream region.

### Definition of TTS

Possible triplex target sequences (TTSs) were defined as polypurine tracks of any size and in any strand. No mismatching in the triplexes was allowed, implying that a strict triplex definition was used. The number of TTSs would increase substantially if 5% or 10% mismatching were allowed.

### Background models of TTS distributions

In order to determine the significance of a given TTS distribution we need to create background distributions. We used first an analytical binomial model [26] where all base dimmers have the same probability. To generate a more reliable background model we modified the method to account for the dimmer-distributions in human genome and also on human promoter (where Pur-Pyr, Pur-Pur and Pyt-Pur are not equally probable). We consider also a numerical background model (that at the dimmer level fits to the binomial model; [see Figure 2 in reference 26]) which allows us to introduce also trimer-biased in the promoter region. This numerical model was build by creating a 10^8^-mer sequence selected which maintain the trimer (or dimer) population found in human promoters. Finally, a last random model (for promoter region) was created by using promoter-specific suffleseq models created with the EMOSS package version 3.0 [30]. In the later case we generate 10 sets of sequences shuffling our promoter collections. A simple visual inspection shows that the real and background distributions are very different, but in any case, we confirm this by running *Clover *[31], a tool for detection of functional DNA motifs via statistical over-representation. For this purpose we create a matrix (length 15-nt), where for all positions A,G scored 1 and T,C score 0 (clover automatically scans both strands).

### TTSs in promoters conserved in human and mouse

To evaluate the conservation of TTSs in the promoter (TTS_p_) region, we took *upstream5000 *file to build 33 mouse assemblies [28]. In order to match human and mouse promoter regions, we translated gene code to protein name using the *loc2ref *file from the NCBI database [32] for both human and mouse genes. We then searched for correspondences in *HomoloGene *Build 39.1 from the NCBI database. This procedure generated a list of 5000 pairs of human-mouse genes.

We calculated human-mouse identity for a chosen 15-nt sequence (TTS or not) from a human region, aligning it across the corresponding mouse region. The alignment was done using a bit-vector alignment algorithm [33]. For each entry, the greatest percentage of matching bases in the best alignment was processed. We estimated the conservation by averaging this value for all region entries.

For the shake of completeness the comparison was also performed using human/mouse aligned sequences present in the UCSC multiz8way (8 vertebrates) multiple alignments. As before conservation is computed by analyzing identity conservation in 15-mer sequences, averaging the data for all the 15-mer windows in the studied segment. Results obtained with this or the previous alignment protocol are very similar, reinforcing the quality of our results. Using these alignments we computed also the conservation in promoter regions annotated as transcription factor binding sites [34,35].

### Functional annotation of groups of genes

To test whether a group of genes was significantly enriched in one or more functional terms (out of several thousands) with respect to the background (usually the rest of genes), we used the FatiGO algorithm [36] from the Babelomics suite [37] for functional annotation of sets of genes. This algorithm uses known functional annotations for genes obtained from the Gene Ontology (GO) consortium databases [38]. Both lists of genes (the group of interest and the background) were converted into two lists of GO terms using the corresponding gene-GO association table. For each GO term the data are represented as a 2 × 2 contingency table with rows representing presence/absence of the GO term, and each column representing each of the two lists. A Fisher's exact test for 2 × 2 contingency tables was used. Since thousands of GO terms are simultaneously tested without an *a priori *hypothesis on any particular term, p-values must be corrected for multiple testing. For this correction, we used the strict false discovery rate (FDR) method described by Benjamini and Yekutieli [39],

### Hypothetical human auto-regulated TTSs

Hypothetical auto-regulated TTSs are those that appear in transcribed and promoter (200 nts upstream) regions of two distinct genes. The TFO able to recognize a TTSp is defined as the sequence that matchs with i) the complement of the TTS or ii) the reverse of the TTS. First case maps potential endogenous TFO for parallel triplex whether the second one maps the potential antiparallel TFOs [see Figure 1]. Genes were divided in three sets: i) containing TTS in the promoter region, ii) containing the corresponding TFO in the transcribed region and iii) other genes (used as background). We ran FATIGO [36,37] to identify possible relationships between genes containing TTS in the promoter and those containing the corresponding TFO in transcribed regions.

### TTSs in transcription factor binding sites

The 0–200 nt region (specially the 0–100 segment) is expected to be largely enriched in transcription factor binding sites. We located all the putative transcription factor binding sites in these region of the human genome by mapping the last public version of the TRANSFAC database [34] to the *upstream5000 *file in the UCSC-Genome Database [28] using the TFBS Perl module [35]. We then computed the average percentage of nucleotides in a TTS with a length of 10 or greater as moving apart the centre of the transcription factor recognition sequence.

### Physical descriptors of DNA

DNA curvature calculation can be done using the data and the algorithm developed by Bolshoy and co-workers [40]. This algorithm calculates the three-dimensional path of a DNA molecule and estimates the curvature of the axis path. The scale is in arbitrary curvature units (c.u.), ranging from 0 (e.g. no curvature) to 1.0, which is the curvature of DNA when wrapped around the histone core of nucleosome.

To predict the stability of the sequence, we used the base step data from Santalucia *et al*. and the formula described in their study [41]. DNA stacking energies are predicted using the accurate interaction energies published for Sponer *et al*. [42,43] for nucleic acid base pairs.

The flexibility of a track was measured by the configurational volume [see eq. 1 in reference 44] in function of the Twist-twist, Shift-shift and Roll-roll force constants determined from MD simulations by Lankas *et al*., [45].

V=(kT)3Ktwist×Ktilt×Kroll     eq. 1
 MathType@MTEF@5@5@+=feaafiart1ev1aaatCvAUfKttLearuWrP9MDH5MBPbIqV92AaeXatLxBI9gBaebbnrfifHhDYfgasaacH8akY=wiFfYdH8Gipec8Eeeu0xXdbba9frFj0=OqFfea0dXdd9vqai=hGuQ8kuc9pgc9s8qqaq=dirpe0xb9q8qiLsFr0=vr0=vr0dc8meaabaqaciaacaGaaeqabaqabeGadaaakeaacqWGwbGvcqGH9aqpdaGcaaqaamaalaaabaGaeiikaGIaem4AaSMaemivaqLaeiykaKYaaWbaaSqabeaacqaIZaWmaaaakeaacqWGlbWscqWG0baDcqWG3bWDcqWGPbqAcqWGZbWCcqWG0baDcqGHxdaTcqWGlbWscqWG0baDcqWGPbqAcqWGSbaBcqWG0baDcqGHxdaTcqWGlbWscqWGYbGCcqWGVbWBcqWGSbaBcqWGSbaBaaaaleqaaOGaaCzcaiaaxMaacqqGLbqzcqqGXbqCcqGGUaGlcqqGGaaicqaIXaqmaaa@5504@

where *k *is Boltzman constant, *T *is the temperature (taken as 298 K) and *Ktwist*, *Ktilt *and *Kroll *are harmonic force constants expressed in kcal/mol • deg^2^

Described methods are implemented in a Perl script library, witch is available upon request.

## Authors' contributions

JRG performed most of the analysis presented in this paper. JMV performed the GO-analysis, with the help and advice of JD. MO performed the analysis of data, directed the study and wrote the paper.

## Supplementary Material

Additional File 1 Frequencies of nucleotides forming part of TTSs for different lengths in the human genome and for random models. Genome, promoter and promoter shuffled data is the same as in Figure 2. Genome and promoter random are computed using a numerical method [see *Methods*] that maintains the trimer (or dimer) population. Click here for file

Additional File 2 A) Percentage of genes with an annotated CpG island in promoter region for a given GO term B) Differential GO-analysis for Genes with TTS of length 20 at the 100 upstream region of promoter. Left panel for the bulk of genes (identical to profiles (100-b) in Figure 5, middle for genes with CpG island, and right for genes without CpG genes. Click here for file

Additional File 3 Percentage of human-mouse identity of 15-nt fragments in several promoter regions for TTS and non-TTS segments of the same size in regulatory region. Alignments used here (difference with Figure 7) were taken from UCSC multiz8way data file. TFBS line show data for predicted transcription factor binding sites [see *Methods*] in every region. 100 non-overlapping random sampling of non-TTS set is computed to calculate error bars. Click here for file
