# Supplementary figures and images for: Exploring the reasons for the large density of triplex-forming oligonucleotide target sequences in the human regulatory regions
Source: BMC Genomics. 2006 Mar 27;7:63. doi: 10.1186/1471-2164-7-63 (PMC1435886; doi:10.1186/1471-2164-7-63)

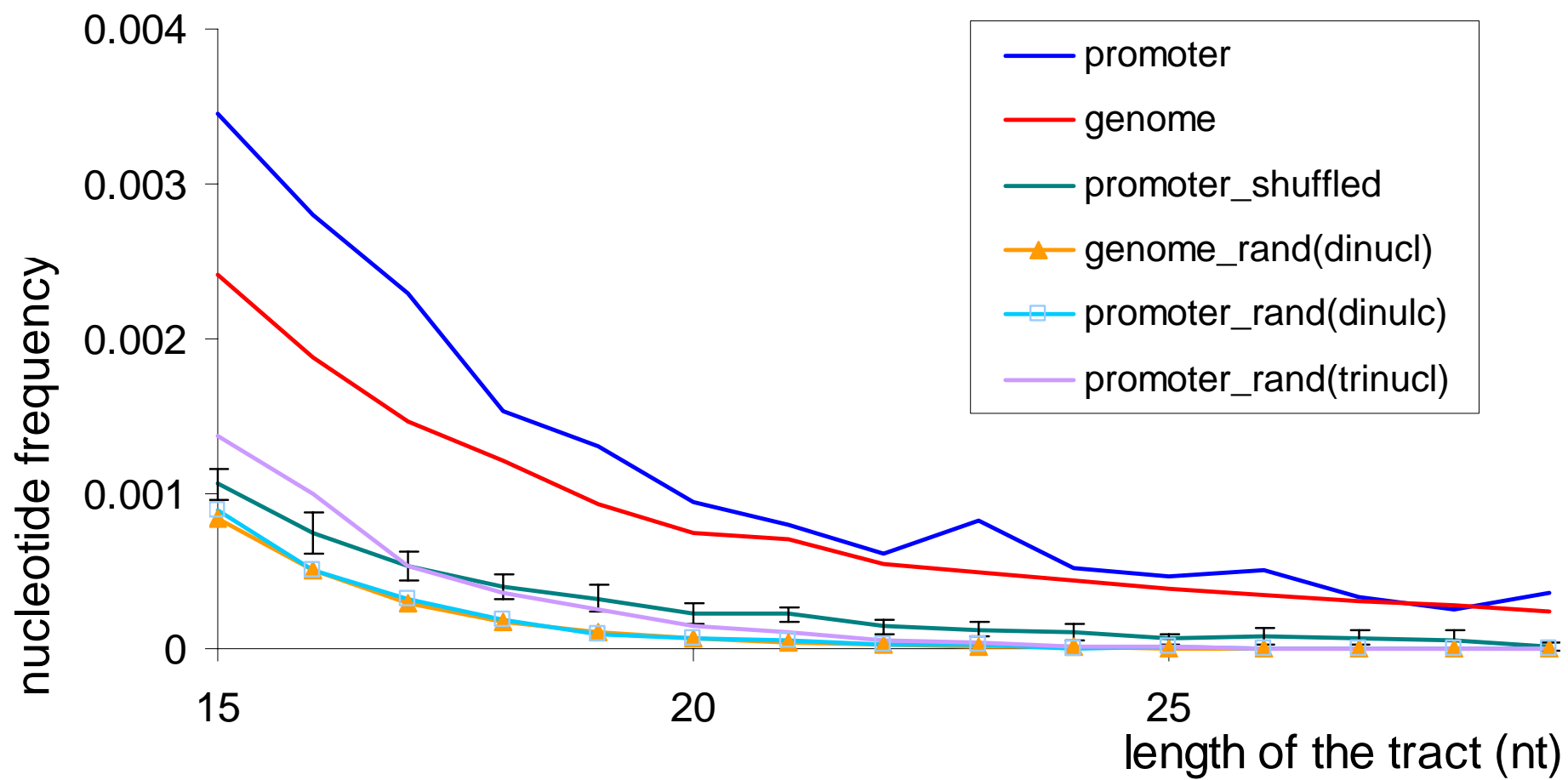

Supplement: Additional File 1 — Frequencies of nucleotides forming part of TTSs for different lengths in the human genome and for random models. Genome, promoter and promoter shuffled data is the same as in Figure 2. Genome and promoter random are computed using a numerical method [see Methods] that maintains the trimer (or dimer) population. [file 1471-2164-7-63-S1.pdf]

A)

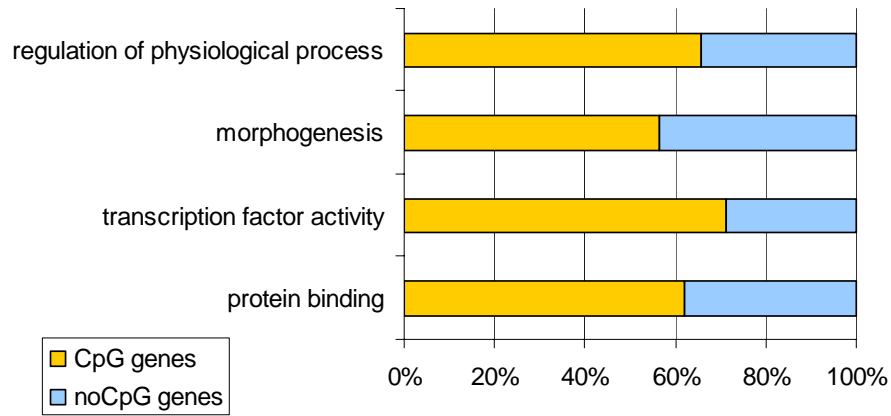

B)

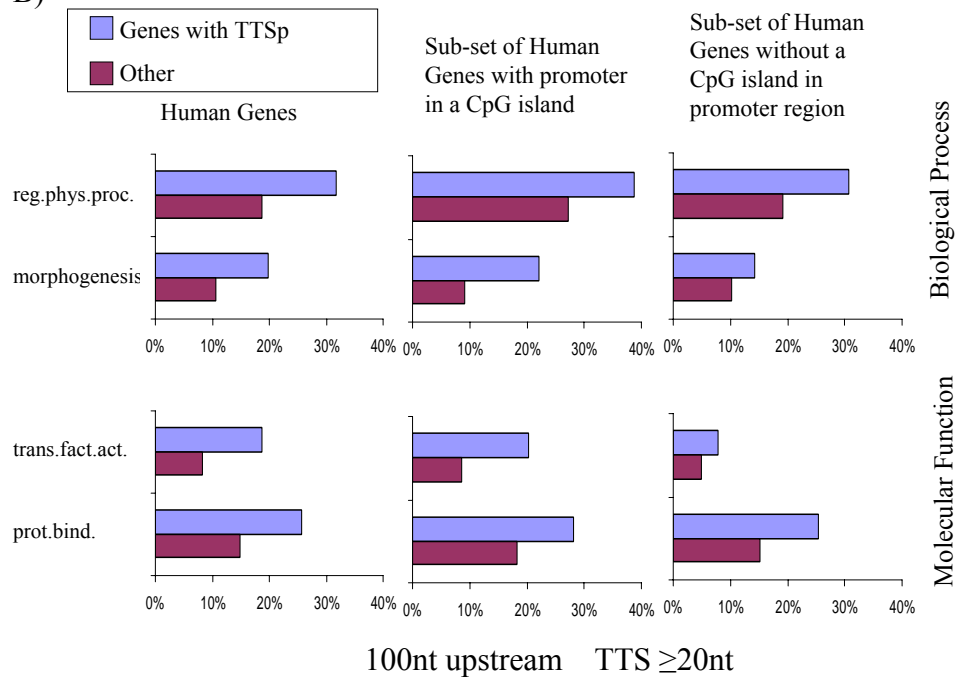

Supplement: Additional File 2 — A) Percentage of genes with an annotated CpG island in promoter region for a given GO term B) Differential GO-analysis for Genes with TTS of length 20 at the 100 upstream region of promoter. Left panel for the bulk of genes (identical to profiles (100-b) in Figure 5, middle for genes with CpG island, and right for genes without CpG genes. [file 1471-2164-7-63-S2.pdf]

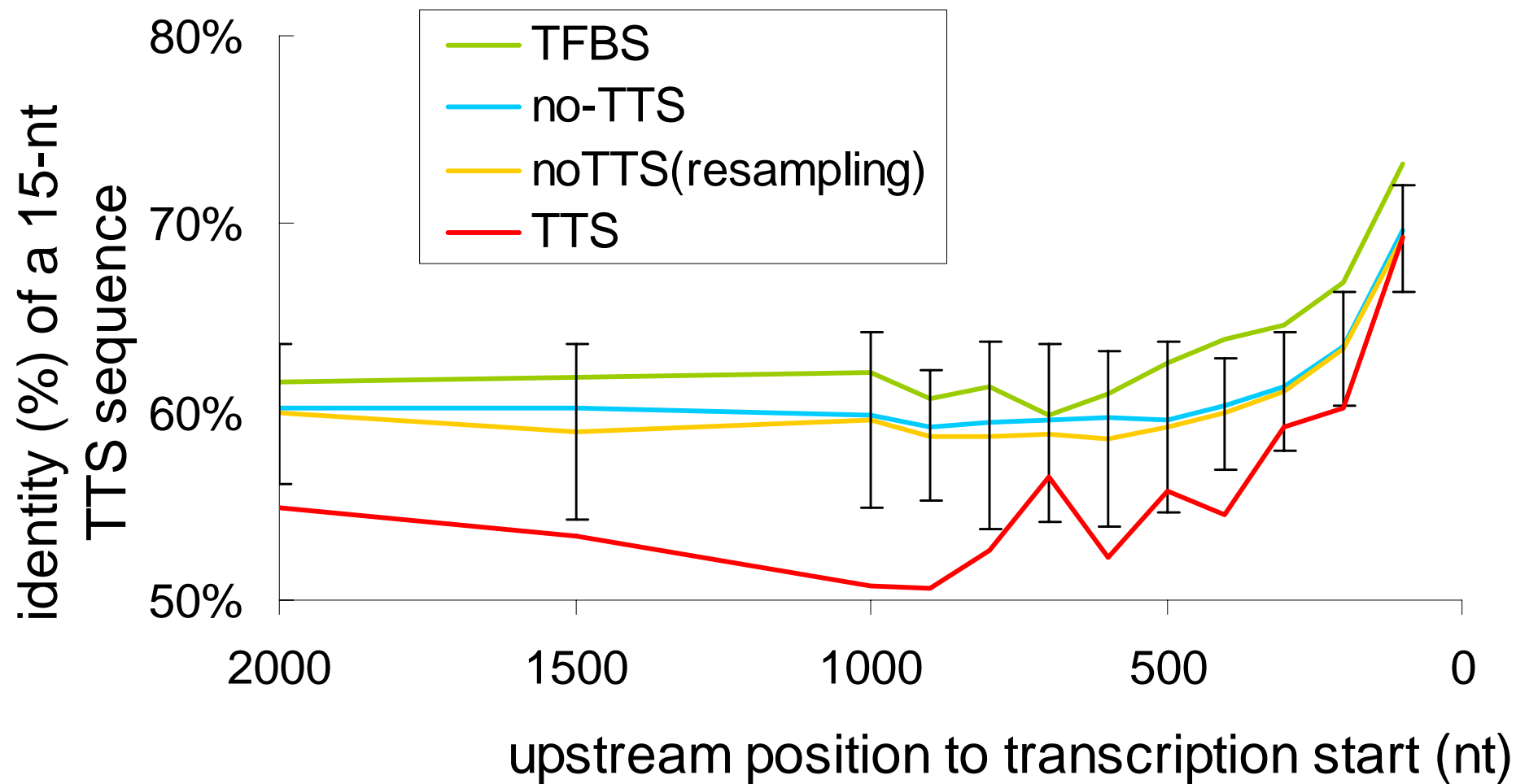

Supplement: Additional File 3 — Percentage of human-mouse identity of 15-nt fragments in several promoter regions for TTS and non-TTS segments of the same size in regulatory region. Alignments used here (difference with Figure 7) were taken from UCSC multiz8way data file. TFBS line show data for predicted transcription factor binding sites [see Methods] in every region. 100 non-overlapping random sampling of non-TTS set is computed to calculate error bars. [file 1471-2164-7-63-S3.pdf]
